# Social activities and different intensities of physical activity among older adults in China: a population-based cohort study

**DOI:** 10.3389/fpubh.2025.1530895

**Published:** 2025-01-29

**Authors:** Song Yang, Qi Zhang, Anhui Zhao, Dongye Lyu

**Affiliations:** ^1^Department of Sports Journalism and Management, Henan Sport University, Zhengzhou, China; ^2^Shandong Polytechnic, Jinan, China; ^3^School of Physical Education (Main Campus), Zhengzhou University, Zhengzhou, China; ^4^College of Education Sciences, The Hong Kong University of Science and Technology (Guangzhou), Guangzhou, China

**Keywords:** physical activity, population aging, social activities, sport, Chinese older adults

## Abstract

**Introduction:**

Population aging is an inevitable consequence of demographic transition and an important issue for human society in the 21st century. Physical activity is widely recognized as a critical factor for improving health, yet the specific impact of different intensities of physical activity on the health of older adults remains underexplored.

**Methods:**

This study addresses this gap by analyzing data from the China Health and Retirement Longitudinal Survey (CHARLS), a nationally representative survey of a cohort of Chinese people (aged ≥45 years) from 150 counties or districts and 450 villages or urban communities across 28 provinces, who were selected by use of multistage stratified probability-proportionate-to-size sampling. The study employed a regression model aiming to analyse the effect of different intensities of physical activity on the health level of the elder adults.

**Results:**

The results demonstrate that physical activity at all intensity levels—high, moderate, and low—significantly improves the health of older adults, with correlation coefficients of −0.245 (*p* < 0.01), −0.080 (*p* < 0.05), and −0.077 (*p* < 0.10), respectively. Among these, high-intensity physical activity is the most effective in enhancing health outcomes. The result further identifies social activities as a mediating factor in this relationship. Moreover, high-intensity exercise proves to be more beneficial for older adults residing in rural areas compared to their urban counterparts.

**Discussion:**

This study demonstrates that engagement in physical activity at all intensity levels—high, moderate, and low—substantially enhances the health of older adults, with high-intensity physical activity demonstrating the most significant impact on health outcomes. Furthermore, the research identifies social activities as a mediating factor in this relationship, highlighting the importance of social engagement in conjunction with physical activity. This suggests that interventions promoting both physical activity and social interaction can be particularly effective in improving the health and wellbeing of the ageing population.

## 1 Introduction

Population aging is an inevitable consequence of demographic transition and an important issue for human society in the 21st century. Aging refers to the increasing proportion of a country’s population aged 65 years and over (1). Since 1865, when France became the first country to enter an aging society, more and more countries in Europe, East Asia and other regions have entered the ranks of the aging. According to United Nations statistics, it is expected that by 2050, more than 1.5 billion people worldwide will enter the 65-and-over age group, especially as the baby boomer generation enters the older adult age group (2). The rapid growth of the global aging population is putting enormous pressure on public healthcare systems and even social security systems (3). How to effectively respond to population aging has become a global concern, and despite the different characteristics and trends of aging in different countries and regions.

China is a populous country with rapid changes in the population age structure and is on track to become one of the most significant nations with an aging population (4). The proportion of people aged 60 and older in China surged from 13.26% in 2010 to 18.70% in 2021, and it is projected to reach 28.0%—equating to 402 million people—by 2040 (5, 6). As China’s population continues to age amid limited social resources, essential social support and attention for older adults may fall short. This shortfall could lead to a rising prevalence of mental health concerns and healthcare challenges among this demographic (7). In response to an aging population, the Chinese government has adopted the World Health Organization’s (WHO) “healthy aging” framework, which promotes a process of aging that is healthy, active, independent, and participatory (8).

A comprehensive understanding of aging is crucial. Physical aging, a natural process inherent to all living organisms, involves multidimensional changes across physiological, psychological, and social domains, reflecting an inevitable law of nature. To sustain or improve the quality of life during aging, adopting diverse and effective strategies is essential. Research indicates that physical activity is among the most recommended non-pharmacological approaches to enhance wellbeing in older adults (9). Consistent engagement in physical activity (PA) offers numerous health advantages, including enhanced muscular and cardio-respiratory fitness, as well as healthy bone development (10). In particular, strength training has emerged as a highly effective intervention. Evidence suggests that it not only enhances muscle strength and balance but also reduces the risk of falls, alleviates the fear of falling, and improves overall physical health. Collectively, these benefits contribute significantly to maintaining autonomy and improving the quality of life in older populations (11). Consequently, fostering active engagement in physical activity among older adults is recognized as a crucial strategy to develop a population that remains healthy, active, and productive as its ages (12).

A growing body of literature suggests an intrinsic link between physical activity and overall health (13, 14). This connection extends beyond the treatment and rehabilitation of physical ailments (15), contributing significantly to the enhancement of psychological and mental wellbeing (16, 17), 2applicable to both young people and adults (18, 19). For example, Ibsen et al. (20) analyzed the effects of various types of physical activity on both physical and mental health outcomes, while Janssen et al. (21) found notably lower crude and adjusted probabilities of experiencing mental health issues across all types of physical activity. Additionally, a study of adolescents in Ireland indicates that participation in higher-intensity physical activity effectively promotes psychological wellbeing (22). Evidence further suggests that the timing and frequency of physical activity, even scheduled throughout a 24-h period, can influence health outcomes (23, 24).

Sports participation serves as a communal activity that fosters social connections and support systems. For example, engagement in sports teams satisfies the fundamental human need for social interaction and affiliation (25). Participation in sports is an effective way to gain peer recognition (26), Physical exercise, with its inherent social interaction properties, provides opportunities for individuals to form more stable social groups through participation in diverse sports programs. Regardless of gender, physical activity with peers may lead to friendships by fulfilling personal socialization needs and fostering a sense of belonging, offering a platform for social development and emotional stability (27, 28). Extensive research has demonstrated that social activities, as a component of a healthy lifestyle, positively influence the physical and mental health of older adults. Participation in these activities not only alleviates depression but also reduces disability rates and enhances overall health and quality of life (29–31). Furthermore, the structure of social networks and active social participation have been identified as key predictors in delaying the onset of dementia in older populations (32, 33). In the context of China—a society characterized by strong relational dynamics (34)—social interactions are deeply embedded in the lives of middle-aged and older adults, many of whom reside in the same communities for extended periods. This environment fosters strong interpersonal connections, where developments within the community can profoundly influence individuals. Extensive participation in social activities within such a relational society often amplifies their effectiveness in promoting health and wellbeing.

Research on the importance and necessity of physical and leisure activities has steadily advanced (35, 36), with broad consensus recognizing the positive impact of physical activity on both physical and mental health. However, objective studies focusing specifically on physical activity participation among older adults remain relatively limited. Empirical analyses from low-and middle-income countries and the global South countries are even more necessary. To address this research gap, this study aims to explore the effects of varying intensities of physical activity on the health of older adults, using data from the China Health and Retirement Longitudinal Study (CHARLS). The following research questions are put forward: RQ 1: What is the impact of different intensities of physical activity on the health of older adults?/RQ 2: Does intensity of physical activity have a different impact on the health of older adults in rural/urban areas?/RQ 3: How does social activity, as a mediating variable, affect the relationship between physical activity intensity and the health level of Chinese older adults? We anticipate that the findings will offer valuable insights to inform targeted policy development and support multifaceted interventions aimed at improving the health conditions of older adults in China.

## 2 Methods

### 2.1 Participants and research design

The data utilized in this study are primarily drawn from the China Health and Retirement Longitudinal Study (CHARLS) 2020 database. CHARLS, a nationally representative longitudinal study conducted by Peking University (37), received approval from the Biomedical Ethics Review Committee of Peking University (IRB00001052-11015), and written informed consent was obtained from all participants. The CHARLS survey has been extensively used to examine health trends among older adults (38, 39), covering 150 county-level units, 450 village-level units, and a population of approximately 17,000 individuals from 10,000 households. In this paper, we focus on the older adult population as the core research subject. Through age filtering (≥ 60 years) and exclusion of missing values, an effective sample size of 3,041 was obtained.

### 2.2 Measures

Physical activity was measured mainly based on the participation in physical activity in the China Health and Retirement Longitudinal Study (CHARLS) database. Specifically, the DA032 questionnaire was used as the main source of data on the types of physical activity of different intensities per week, which resulted in three core explanatory variables including high, moderate and low intensity physical activity. According to the questionnaire, High-intensity sports refer to intense activities that make you short of breath, mainly by engaging in aerobics, fast biking, and so on. Moderate-intensity sports are activities that make you breathe faster than usual, such as regular-paced cycling, Tai-chi, and brisk walking, while Low-intensity physical activity encompassed leisure activities, light exercises and walking.

In turn, the different intensity types further distinguish whether to participate in the relevant type of physical activity or not, and finally constitute “0” as not engaging in the relevant intensity of physical activity, and “1” as engaging in the relevant intensity of physical activity. Moreover, the health level of the older adult was measured by the questionnaire DA001 in the CHARLS database, which asked “How do you think your health is? Is it very good, good, fair, poor, or very poor?” as the primary data source, and the respondents’ self-reported criteria for evaluating their own health as the primary data outcome. Where “1” is very good, “2” is good, “3” is fair, and “4” is poor, “5” is very poor. Additionally, Social activity, was measured mainly based on the CHARLS database DA038, Questions such as “Have you carried out the following social activities in the past month?” was formed. The mechanism variable of this study was formed by data cleaning, screening, and summing the items of participation in social activity by respondents’ answers to this item. Higher values represent higher frequency of participation in social activity (Table 1).

**Table 1:** Descriptive statistical analysis.

Variables		*n*	percentage
Domicile	Agricultural	2,474	81.35%
	Non-agricultural	567	18.65%
	66–70	1,539	50.60%
	71–75	873	28.70%
	76–80	448	14.74%
	>80	181	5.94%
Gender	Male	1,564	51.43%
	Female	1,477	48.57%
Marital status	Married	2,298	75.57%
	Unmarried	743	24.43%
Education level	Illiterate	898	29.53%
	Not completing primary school	717	23.58%
	Sishu/Old-style private school	6	0.20%
	Primary school	813	26.73%
	Middle school	413	13.58%
	High school	81	2.66%
	Vocational school	69	2.27%
	Two-/Three-year college/associate degree	26	0.85%
	Four-Year college/bachelor’s degree	18	0.59%
Family size	0	2,113	69.48%
	1	326	10.72%
	2	230	7.56%
	3	159	5.23%
	4	156	5.13%
	5	30	0.99%
	6	15	0.49%
	7	6	0.20%
	8	2	0.07%
	10	1	0.03%
	11	1	0.03%
	14	2	0.07%
Personal income	Have personal income	273	8.98%
	No personal income	2,768	91.02%
Health	Very good	310	10.19%
	Good	324	10.65%
	Fair	1,465	48.17%
	Poor	677	22.26%
	Very poor	265	8.71%
High	Engage in high-intensity physical exercise	898	29.53%
	Didn’t engage in high-intensity physical exercise	2,143	70.47%
Middle	Engage in moderate-intensity physical exercise	1,475	48.50%
	Didn’t engage in moderate-intensity physical exercise	1,566	51.50%
Low	Engage in low-intensity physical exercise	2,326	76.49%
	Didn’t engage in low-intensity physical exercise	715	23.51%

### 2.3 Covariates

Covariates, including sociodemographic information, lifestyle behaviors, and health-related factors, were collected through a structured questionnaire. In this study, we control for variables based on the findings of previous studies and the specific context of our research, including domicile, age, gender, marital status, education level, family size, and personal income. Domicile is a dummy variable, where 0 represents rural households and 1 represents non-agricultural households. Age represents the individual’s actual age, calculated using the survey date and the participant’s birth date. Gender is a dummy variable, with 0 indicating female and 1 indicating male. Marital status is also a dummy variable, where 0 denotes unmarried and 1 denotes married. Education level is measured using the questionnaire’s BA010 item, ranging from 1 to 10, with higher values indicating higher levels of education. Individual health is based on the DA001 item of the questionnaire; self-assessed health levels of “general” and above are coded as 1, with other levels coded as 0. Family size reflects the actual household composition as reported in the questionnaire. Personal income is a dummy variable derived from item GA001, where 1 indicates the presence of personal income and 0 indicates its absence.

### 2.4 Statistical analysis

In this study, statistical analyses of the variables were conducted using STATA16 software. The basic characteristics of the variables were first summarized through descriptive statistical methods. Building upon prior discussions regarding the impact of different types of physical exercise on the health levels of elder individuals, three mathematical models were constructed to analyze the relationships between high, moderate, and low-intensity physical exercise and health outcomes within this group. The ordinary least squares (OLS) method was then used to test and analyze these relationships, with all relevant control variables, including domicile, age, gender, marital status, education, family size, and personal income, accounted for in the analysis. In order to verify the overall robustness of the model, the robustness test analysis is carried out in this section by replacing the baseline regression model. Since the robustness of the Tobit model is relatively dependent on the distribution, if the data do not follow a normal distribution, it may lead to estimation bias. Therefore, OLS with robust standard deviation is directly used in this part to regress the model. The results are shown in Table 2, which shows that the core explanatory variables do not differ significantly from those in the baseline regression model, indicating that the conclusions of this study are robust.

**Table 2:** Robustness test results (replacement of regression model).

	(1)	(2)	(3)
High	−0.257^***^		
	(−5.943)		
Middle		−0.065^**^	
		(−2.118)	
Low			−0.082^*^
			(−1.649)
Control	YES	YES	YES
*N*	3,041	3,041	3,041
*R* ^2^	0.0313	0.0218	0.0213

z statistics in parentheses, ^*^p < 0.10, ^**^p < 0.05, ^***^p < 0.01.

## 3 Results

The central question of this study is to analyse the effect of different intensities of physical activity on the health level of the elder adults, therefore the baseline regression model of this paper is set in this section as follows:


(1) $${\mathrm{Health}}_{i}=\beta_{0}+\beta_{1}{\mathrm{sportexercise}}_{i}+\beta_{2}{\mathrm{control}}_{i}+\varepsilon_{i}$$

Among them, ${\mathrm{Health}}_{i}$ represents the health level indicators of the older adult ${\mathrm{sportexercise}}_{i}$ denotes physical activity indicators, including high-intensity physical activity, moderate-intensity physical activity and low-intensity physical activity (see Table 3). ${\mathrm{control}}_{i}$ denotes control variables, including all control variables listed in the previous section. $\beta_{0}$ is the intercept term, $\beta_{1}$ and $\beta_{2}$ are the coefficients to be estimated, and $\varepsilon_{i}$ is the random perturbation item. The benchmark regression model is processed and analysed using the Tobit model. Table 2 shows the benchmark regression results of this paper. According to the regression results, it can be found that both high-intensity physical exercise, moderate-intensity physical exercise and low-intensity physical exercise can significantly promote the health level of the older adult. Among them, high-intensity physical exercise has a significant positive effect on the health level of the older adult, with a regression coefficient of −0.245 (*p* < 0.01), moderate-intensity physical exercise has a significant positive effect on the health level of the older adult, with a regression coefficient of −0.080 (*p* < 0.05), and low-intensity physical exercise has a significant positive effect on the health level of the older adult, with a regression coefficient of −0.080 (p < 0.05). The regression coefficient is −0.077 (*p* < 0.10), and this effect shows an overall decreasing trend of high intensity-moderate intensity-low intensity.

**Table 3:** Benchmark regression results.

	(1)	(2)	(3)
High	−0.245^***^		
	(−5.856)		
Middle		−0.080^**^	
		(−2.108)	
Low			−0.077^*^
			(−1.721)
Gender	0.066	0.090^**^	0.082^**^
	(1.611)	(2.185)	(2.006)
Marital Status	−0.103^**^	−0.091^*^	−0.089^*^
	(−2.232)	(−1.960)	(−1.923)
Education level	0.024^*^	0.030^**^	0.029^**^
	(1.940)	(2.457)	(2.366)
Family size	0.009	0.006	0.006
	(0.674)	(0.445)	(0.437)
Personal income	−0.391^***^	−0.403^***^	−0.414^***^
	(−5.885)	(−6.046)	(−6.209)
_cons	3.978^***^	3.707^***^	3.681^***^
	(13.290)	(12.444)	(12.380)
*N*	3,041	3,041	3,041
pseudo *R*^2^	0.011	0.008	0.007

z statistics in parentheses, ^*^p < 0.10, ^**^p < 0.05, ^***^p < 0.01.

China’s unique household registration system enables categorization of the study sample into rural and urban older adult groups based on household registration status, allowing for subgroup regression analysis. As indicated in Table 4, high-intensity physical activity significantly enhances health levels in both rural and urban elder populations, with regression coefficients of −0.2504 (*p* < 0.01) for rural areas and −0.1943 (*p* < 0.10) for urban areas. The effect of high-intensity physical activity on rural elder individuals’ health is notably greater than that observed for their urban counterparts. Moderate-intensity physical activity significantly promotes health for the rural older adult showing a regression coefficient of −0.0710 (p < 0.10), though it is not statistically significant for urban older adult health. Although low-intensity physical activity appears to improve health for both rural and urban older adult groups, it did not meet the threshold for statistical significance.

**Table 4:** Heterogeneity analysis based on residence.

	(1) Rural	(2) Urban	(3) Rural	(4) Urban	(5) Rural	(6) Urban
High	−0.2504^***^	−0.1943^*^				
	(−5.4916)	(−1.7706)				
Middle			−0.0710^*^	−0.1191		
			(−1.6540)	(−1.5031)		
Low					−0.0656	−0.1772
					(−1.3644)	(−1.4089)
Marital status	−0.0839	−0.2155^**^	−0.0699	−0.2089^*^	−0.0678	−0.2115^*^
	(−1.6395)	(−1.9914)	(−1.3590)	(−1.9282)	(−1.3194)	(−1.9521)
Education level	0.0332^**^	0.0017	0.0400^***^	0.0075	0.0386^***^	0.0080
	(2.2620)	(0.0809)	(2.7021)	(0.3640)	(2.6196)	(0.3868)
Family size	0.0068	0.0236	0.0042	0.0179	0.0042	0.0176
	(0.4640)	(0.6664)	(0.2847)	(0.5064)	(0.2821)	(0.4963)
Personal income	−0.3970^***^	−0.3209^*^	−0.4118^***^	−0.3223^*^	−0.4228^***^	−0.3119^*^
	(−5.5183)	(−1.7739)	(−5.6898)	(−1.7802)	(−5.8493)	(−1.7211)
_cons	3.6308^***^	3.6678^***^	3.3367^***^	3.7186^***^	3.3257^***^	3.7750^***^
	(10.6039)	(6.1284)	(9.7879)	(6.1131)	(9.7443)	(6.0737)
*N*	2,474	567	2,474	567	2,474	567
pseudo *R*^2^	0.0103	0.0081	0.0065	0.0075	0.0064	0.0074

z statistics in parentheses, ^*^p < 0.10, ^**^p < 0.05, ^***^p < 0.01.

On the other hand, social activity is considered to be an important pathway for physical activity to promote consumption upgrading among the older adult. The main purpose of this section is to empirically test whether this mechanism of action is valid, and this study constructs the following mechanism-testing model:


(2) $${\mathrm{Health}}_{i}=\beta_{0}+\beta_{1}{\mathrm{sportexercise}}_{i}+\beta_{2}{\mathrm{control}}_{i}+\varepsilon_{i}$$

(3) $$M_{i}=\alpha_{0}+\alpha_{1}{\mathrm{sportexercise}}_{i}+\alpha_{2}\;{\mathrm{Control}}_{i}+\varepsilon_{i}$$

(4) $${\mathrm{Health}}_{i}=\varphi_{0}+\varphi_{1}{\mathrm{sportexercise}}_{i}+\varphi_{2}M_{i}+\varphi_{3}{\mathrm{control}}_{i}+\varepsilon_{i}$$

In this study, M represents the mediator variable for social activities, derived from survey responses to question DA038 on social activities. Following data cleaning, filtering, computation, and summation, this social activity variable is used as the mechanism variable for analysis. In the initial benchmark regression model, the coefficient β_1 was found to be significantly positive, indicating that physical activity positively influences the health levels of the older adult. Therefore, this section focuses on coefficients α_1, *φ*_1, and φ_2. If α_1 and φ_2 are significant while φ_1 is not, this suggests a fully mediated effect. If all three are significant, further analysis is needed: if the product of α_1 and φ_2 has the same sign as〖 φ〗_1, it indicates a partial mediation effect (see Table 5).

**Table 5:** Results of mechanism effect tests (mediating variables based on social activities).

	(1)	(2)	(3)	(4)	(5)	(6)
High	0.1187^***^	−0.2402^***^				
	(3.4625)	(−5.7261)				
Middle			0.2516^***^	−0.0685^*^		
			(8.2197)	(−1.7888)		
Low					0.2087^***^	−0.0668
					(5.7655)	(−1.4906)
Social activity		−0.0433^*^		−0.0453^**^		−0.0477^**^
		(−1.9542)		(−2.0175)		(−2.1385)
Marital status	0.0992^***^	−0.0989^**^	0.1005^***^	−0.0864^*^	0.0946^**^	−0.0847^*^
	(2.6214)	(−2.1382)	(2.6807)	(−1.8606)	(2.5116)	(−1.8247)
Education level	0.0742^***^	0.0267^**^	0.0646^***^	0.0329^***^	0.0689^***^	0.0321^***^
	(7.4790)	(2.1860)	(6.5527)	(2.6799)	(6.9637)	(2.6175)
Family size	−0.0070	0.0088	−0.0059	0.0058	−0.0055	0.0057
	(−0.6346)	(0.6523)	(−0.5347)	(0.4253)	(−0.5003)	(0.4175)
Personal income	−0.0398	−0.3925^***^	−0.0535	−0.4058^***^	−0.0215	−0.4150^***^
	(−0.7328)	(−5.9144)	(−0.9927)	(−6.0849)	(−0.3973)	(−6.2289)
_cons	0.7601^***^	4.0113^***^	0.6172^**^	3.7348^***^	0.7310^***^	3.7162^***^
	(3.1018)	(13.3876)	(2.5665)	(12.5322)	(3.0289)	(12.4882)
*N*	3,041	3,041	3,041	3,041	3,041	3,041
pseudo *R*^2^	0.0223	0.0113	0.0294	0.0080	0.0250	0.0079
Sobel test	−0.0079***		−0.0059*		−0.0035	
Indirect effect	−0.0079**		−0.0059***		−0.0035***	
Direct effect	−0.4326**		−0.0452**		−0.04774**	
Total effect	−0.5120**		−0.0512**		−0.0512**	
Proportion of intermediary effect	15.51%		11.56%		6.76%	

z statistics in parentheses, ^*^p < 0.10, ^**^p < 0.05, ^***^p < 0.01.

The regression results demonstrate that high-, moderate-, and low-intensity physical activity all significantly enhance social activity, with coefficients of 0.1187 (*p* < 0.01), 0.2516 (*p* < 0.01), and 0.2087 (p < 0.01), respectively, aligning with social practice observations. Further analysis using the mechanism model confirms social activity as a crucial pathway through which physical activity improves older adult health levels. High-and moderate-intensity physical activity passed the Sobel test, showing coefficients of −0.0079 (p < 0.01) and −0.0059 (*p* < 0.10), with proportion of intermediary effect of 15.51 and 11.56%, respectively.

In addition, considering that different intensities of physical activity may have different effects on older adult groups of different genders, this part is analyzed based on the heterogeneity at the gender level. The group regression results are shown in Table 6. Whether it is high-intensity, moderate-intensity or low-intensity physical exercise, it can effectively improve the health level of men and women in the older adult. Among them, high-intensity physical exercise has a significant health-promoting effect on the older adult male and female groups (*p* < 0.01), but it has a higher effect on the older adult male group, with a regression coefficient of −0.2775 (*p* < 0.01). Moderate-intensity physical exercise can significantly improve the physical health of the older adult male group, with a regression coefficient of −0.0893 (*p* < 0.10), while the health-promoting effect on the older adult female group has not passed the significance test. Low-intensity physical exercise can significantly improve the physical health of the older adult female group, with a regression coefficient of −0.1285 (*p* < 0.05), while the health-promoting effect on the older adult male group has not passed the significance test. The possible reason for this result is that due to the unique physical differences between men and women, there are certain differences in the effects of physical exercise of different intensities on their health levels.

**Table 6:** Results of mechanism effect tests (mediating variables based on gender differences).

	(1) Male	(2) Female	(3) Male	(4) Female	(5) Male	(6) Female
High	−0.2775^***^	−0.2102^***^				
	(−5.0286)	(−3.2904)				
Middle			−0.0893^*^	−0.0683		
			(−1.7415)	(−1.2147)		
Low					−0.0197	−0.1285^**^
					(−0.3129)	(−2.0313)
Marital status	−0.1938^***^	−0.0550	−0.1684^**^	−0.0501	−0.1646^**^	−0.0488
	(−2.7223)	(−0.8849)	(−2.3553)	(−0.8030)	(−2.3016)	(−0.7827)
Education level	0.0165	0.0316^*^	0.0253	0.0359^*^	0.0232	0.0362^*^
	(1.0551)	(1.6600)	(1.6111)	(1.8668)	(1.4782)	(1.8885)
Family size	0.0095	0.0063	0.0074	0.0028	0.0064	0.0025
	(0.5047)	(0.3217)	(0.3887)	(0.1419)	(0.3388)	(0.1283)
Personal income	−0.4024^***^	−0.3908^***^	−0.4151^***^	−0.4040^***^	−0.4263^***^	−0.4120^***^
	(−5.1231)	(−3.2292)	(−5.2419)	(−3.3300)	(−5.3904)	(−3.3999)
_cons	4.4955^***^	3.7499^***^	4.2192^***^	3.5463^***^	4.1432^***^	3.5631^***^
	(11.2266)	(8.9384)	(10.5476)	(8.4269)	(10.3424)	(8.6010)
*N*	1,564	1,477	1,564	1,477	1,564	1,477
pseudo *R*^2^	0.0152	0.0070	0.0103	0.0049	0.0096	0.0055

z statistics in parentheses, ^*^p < 0.10, ^**^p < 0.05, ^***^p < 0.01.

## 4 Discussion

This study examines the impact of varying levels of physical activity intensity on health outcomes among older adults in China. The findings indicate that physical activity, particularly high-intensity exercise, significantly contributes to health condition in this population. These results align with previous research, which suggests that both high and moderate-intensity exercise can enhance the health of older adults in diverse contexts (40), including developed nations like the United States and developing countries such as China. Furthermore, the study reveals that the health benefits of exercise are not uniform across different demographics; specifically, high-intensity exercise proves to be more beneficial for older adults residing in rural areas compared to their urban counterparts. This disparity can be attributed to China’s unique urban–rural context, where lifestyles significantly differ for the older adult. Rural older adults often engage in longstanding habits of physical labor, which inherently combine exercise with daily leisure activities, thereby promoting better physical health. Conversely, the urban older adult, who typically experience higher-quality living environments and predominantly engage in non-physical labor, may struggle to adapt to high-intensity physical activities.

The analysis also identifies social activity as a mediating factor between physical activity and health outcomes. Social activity performed with proportion of intermediary effect of 15.51 and 11.56% of the health condition associated with high-and moderate-intensity activities, respectively. Participation in sports and physical activities provides Chinese older adults with valuable opportunities for social engagement, which in turn enhances their health. These findings corroborate previous studies linking increased social activity to reduced mortality risk among older adults (40), as well as demonstrating that enhanced physical activity intensity can alleviate depressive symptoms and improve overall health (41). In light of these findings, it is imperative for policymakers and stakeholders to promote physical activity among the older adult. Such initiatives can help mitigate loneliness and emotional challenges faced by older adults, facilitate social participation, and ultimately enhance their quality of life. Additionally, the results underscore the pressing social equity issue related to the stark disparities in urban and rural development in China. Evidence from more developed regions indicates that economic growth positively influences the amount of time individuals dedicate to physical activity (42), with long-term participation in physical activity showing a positive correlation with GDP and overall affluence (43). Data specific to China further illustrate a link between real GDP per capita and levels of physical activity, suggesting that individuals in economically developed regions engage in more physical activity compared to those in less developed areas (44).

The findings of this study present several practical implications. First, the study underscores the positive impact of physical activity on the health of older adults. This finding highlights the importance of promoting physical activity as a means to enhance the wellbeing and health outcomes of aging populations. Second, the selection of China as the study sample is particularly valuable. As a multiethnic and multicultural nation, China offers a diverse research context. Furthermore, as a rapidly developing country with a substantial and growing older adult population, the focus on the health and wellbeing of this demographic is both timely and critical. Third, the study highlights the role of gender differences in older adults’ participation in sports and the associated effects on health. While the results do not solely attribute these differences to biological factors, they draw attention to the influence of unequal opportunities for sports participation and habit formation, shaped by historical and social contexts. Although the observed gender differences are not pronounced, they contribute to broader discussions on gender equity and physical activity, offering important insights for emerging research on gender and sports.

This study underscores the importance of selecting appropriate physical activity for older adults, providing valuable guidance for future physical activity recommendations. However, several limitations must be acknowledged. Firstly, the use of cross-sectional data restricts the ability to infer causal relationships between physical activity and health outcomes. Longitudinal studies would be advantageous in establishing temporal connections and understanding how physical activity influences health over time. Secondly, the reliance on self-report methods may introduce subjective biases. While self-reported measures of physical activity, social activities, and health levels are common in brief surveys, they may not accurately capture participants’ actual lifestyles and social behaviors. Furthermore, the broad categorization of physical activity intensity and the generalized measurement of health levels could diminish the precision of the findings. Future research should address these concerns by adopting longitudinal designs and utilizing more objective monitoring methods, such as wearable fitness trackers or clinical health assessments, to enhance the accuracy and reliability of data regarding older adults’ physical activity levels and their impact on health conditions. Additionally, due to the limitations inherent in the dataset—specifically, the absence of a systematic summary and analysis of various categories of sports—result in an oversight of the nuanced distinctions among different forms of physical exercise, thereby creating gaps in the current findings. Future studies should aim to explore the specific effects of different types of physical activities across varying intensity levels to provide a more comprehensive understanding of their impacts on health while identifying which specific sports are most suitable for older adults. Coupled with enhanced data collection efforts, future studies can examine the health impacts of diverse types of physical activities and sports across different age groups. Additionally, physical activity research is critical in addressing social equity and promoting inclusiveness, particularly across diverse age groups. Future research should prioritize these dimensions to ensure broader applicability and relevance of findings. This includes examining how different socio-economic backgrounds, cultural contexts, and access to resources influence the relationship between physical activity and health in older populations.

## 5 Conclusion

This study asserts that engagement in physical activity at all intensity levels—high, moderate, and low—substantially enhances the health of older adults, with high-intensity physical activity demonstrating the most significant impact on health outcomes. Furthermore, the research identifies social activities as a mediating factor in this relationship, highlighting the importance of social engagement in conjunction with physical activity. These findings emphasize the critical role of physical activity in improving health conditions among older adults, underscoring the necessity for public health practitioners and policymakers to promote appropriate levels of physical activity and social participation within this demographic. In light of these findings, we propose several interventions aimed at improving the quality of life for older adults through enhanced sports participation: (1) Increase the availability of sports facilities and diversify the range of sports activities to facilitate greater participation among older adults; (2) Develop and implement moderate-and high-intensity sports programs that are specifically tailored to the characteristics and needs of older adults, thereby providing effective pathways for their engagement in physical activity; (3) Encourage the establishment of social sports organizations for older adults, creating supportive platforms for social interaction and community building within this population; (4) Emphasize the provision of sports programs in rural areas while also developing urban sports initiatives, with particular attention to the participation of older adult individuals in rural contexts. These recommendations call for the development of tailored intervention strategies that consider the unique needs of specific demographic groups, particularly in relation to varying levels of socioeconomic development. Furthermore, research on physical activity should adopt a proactive approach to address social equity issues and foster inclusivity, prioritizing the wellbeing of all individuals.

## Data Availability

The original contributions presented in the study are included in the article/supplementary material, further inquiries can be directed to the corresponding author.

## References

[1] Álvarez-García J Durán-Sánchez A Del Río-Rama MDLC García-Vélez DF. Active ageing: mapping of scientific coverage. IJERPH. (2018) 15:2727. doi: 10.3390/ijerph15122727, PMID: 30513943 PMC6313563

[2] Morato J Sanchez-Cuadrado S Iglesias A Campillo A Fernández-Panadero C. Sustainable technologies for older adults. Sustain For. (2021) 13:8465. doi: 10.3390/su13158465

[3] Luo Y Waite LJ. Loneliness and mortality among older adults in China. J Gerontol Ser B Psychol Sci Soc Sci. (2014) 69:633–45. doi: 10.1093/geronb/gbu007, PMID: 24550354 PMC4049147

[4] Fan S Hui L Dou L Hui M. Impact of population ageing and declining birth rates on household consumption structure: evidence from China. Financ Res Lett. (2024) 68:105932. doi: 10.1016/j.frl.2024.105932

[5] Ning Jizhe DHOT. Main data of the seventh National Population Census News Release. Xicheng: National Bureau of Statistics of China (2023).

[6] Che B Zheng X Chen B Lu Y Zhang Y Xu B. The temporal trend of tuberculosis burden in an ageing population in China: a secondary data analysis from the GBD 2019. BMC Pulm Med. (2024) 24:476. doi: 10.1186/s12890-024-03293-2, PMID: 39334014 PMC11437723

[7] Yu Q Ren Y Wu J. Loneliness shapes disparities in healthy life expectancy: a multi-state analysis from China. BMC Public Health. (2024) 24:1492. doi: 10.1186/s12889-024-18975-z, PMID: 38834967 PMC11514865

[8] Yu Y. Healthy ageing in urban China: governing the ageing population. Geogr J. (2021) 187:28–38. doi: 10.1111/geoj.12372

[9] Parra Rizo MA. Efecto y adecuación del ejercicio para la mejora cardiovascular de la población mayor de 65 años. PSSA. (2020) 8:670. doi: 10.21134/pssa.v8i1.670

[10] Hao Y Lyu D Zhang S Guo B Yan J. Sports participation and depressive symptoms in youth: demographic differences. IJMHP. (2024) 26:865–73. doi: 10.32604/ijmhp.2024.055231

[11] Rivera Miranda P Trujillo Altamirano C Yáñez Yáñez R McArdle Draguicevic N Quintana Peña P Parra Rizo MA. Entrenamiento de fuerza para prevención de caídas en personas mayores: Una revisión sistemática. SUN. (2024) 40:216–38. doi: 10.14482/sun.40.01.650.452

[12] Alvarez-Lourido D Paniza Prados JL Álvarez-Sousa A. Ageing, leisure time physical activity and health in Europe. Healthcare. (2023) 11:1247. doi: 10.3390/healthcare11091247, PMID: 37174789 PMC10178047

[13] Heinrich KM Maddock J Bauman A. Exploring the relationship between physical activity knowledge, health outcomes expectancies, and behavior. J Phys Act Health. (2011) 8:404–9. doi: 10.1123/jpah.8.3.404, PMID: 21487140

[14] Marks-Vieveen JM Uijtdewilligen L Motazedi E Stijnman DPM Van Den Akker-Scheek I Bouma AJ. Physical activity levels, correlates, and all-cause mortality risk in people living with different health conditions. J Phys Act Health. (2024) 21:394–404. doi: 10.1123/jpah.2023-0387, PMID: 38402878

[15] Plummer LC Chalmers KA. Health literacy and physical activity in women diagnosed with breast cancer. Psycho-Oncology. (2017) 26:1478–83. doi: 10.1002/pon.4318, PMID: 27859877

[16] Rodríguez-Romo G Acebes-Sánchez J García-Merino S Garrido-Muñoz M Blanco-García C Diez-Vega I. Physical activity and mental health in undergraduate students. IJERPH. (2022) 20:195. doi: 10.3390/ijerph20010195, PMID: 36612516 PMC9819335

[17] Happell B Platania-Phung C Scott D. Placing physical activity in mental health care: a leadership role for mental health nurses: PHYSICAL ACTIVITY IN MENTAL HEALTH CARE. Int J Ment Health Nurs. (2011) 20:310–8. doi: 10.1111/j.1447-0349.2010.00732.x, PMID: 21896121

[18] Halliday AJ Kern ML Turnbull DA. Can physical activity help explain the gender gap in adolescent mental health? A cross-sectional exploration. Ment Health Phys Act. (2019) 16:8–18. doi: 10.1016/j.mhpa.2019.02.003

[19] Maher JP Pincus AL Ram N Conroy DE. Daily physical activity and life satisfaction across adulthood. Dev Psychol. (2015) 51:1407–19. doi: 10.1037/dev0000037, PMID: 26280838 PMC4579061

[20] Ibsen B Elmose-Østerlund K Høyer-Kruse J. Associations of types of physical activity with self-rated physical and mental health in Denmark. Prev Med Rep. (2024) 37:102557. doi: 10.1016/j.pmedr.2023.102557, PMID: 38205166 PMC10776653

[21] Janssen I Campbell JE Zahran S Saunders TJ Tomasone JR Chaput J-P. Timing of physical activity within the 24-hour day and its influence on health: a systematic review. Health Promot Chronic Dis Prev Can. (2022) 42:129–38. doi: 10.24095/hpcdp.42.4.02, PMID: 35481335 PMC9116725

[22] Molcho M Gavin A Goodwin D. Levels of physical activity and mental health in adolescents in Ireland. IJERPH. (2021) 18:1713. doi: 10.3390/ijerph18041713, PMID: 33578906 PMC7916674

[23] Shi G Liang C Zang W Bao R Yan J Zhou L. 24-hour movement behaviours and self-rated health in Chinese adolescents: a questionnaire-based survey in eastern China. PeerJ. (2023) 11:e16174. doi: 10.7717/peerj.16174, PMID: 37842041 PMC10576499

[24] Zulyniak S Williams JV Bulloch AG Lukmanji A Patten SB. Physical activity and mental health: a cross-sectional study of Canadian youth. J Can Acad Child Adolesc Psychiatry. (2020) 29:241–52. PMID: 33184568 PMC7595261

[25] Spaaij R Knoppers A Jeanes R. “We want more diversity but…”: resisting diversity in recreational sports clubs. Sport Manage Rev. (2020) 23:363–73. doi: 10.1016/j.smr.2019.05.007

[26] Daniels E Leaper C. A longitudinal investigation of sport participation, peer acceptance, and self-esteem among adolescent girls and boys. Sex Roles. (2006) 55:875–80. doi: 10.1007/s11199-006-9138-4

[27] Lee S Shin M Smith AL. The relationship of physical activity from physical education with perceived peer acceptance across childhood and adolescence. J Sch Health. (2019) 89:452–9. doi: 10.1111/josh.12756, PMID: 30916416

[28] Eather N Wade L Pankowiak A Eime R. The impact of sports participation on mental health and social outcomes in adults: a systematic review and the ‘mental health through sport’ conceptual model. Syst Rev. (2023) 12:102. doi: 10.1186/s13643-023-02264-8, PMID: 37344901 PMC10286465

[29] He FK Zhou YS Xie SH. Study on the relationship between leisure life style and health of the elderly. Modern Prev Med. (2024) 51:142. doi: 10.20043/j.cnki.MPM.202307397

[30] Chen S Yu YF Zhang YM. Study on the current status and influencing factors of social activities among elderly patients with chronic diseases in China. J Mudanjiang Med Univ. (2022) 43:162–6. doi: 10.13799/j.cnki.mdjyxyxb.2022.04.001

[31] Morrow-Howell N Hong S-I Tang F. Who benefits from volunteering? Variations in perceived benefits. Gerontologist. (2009) 49:91–102. doi: 10.1093/geront/gnp007, PMID: 19363007

[32] Berkman LF. Which influences cognitive function: living alone or being alone? Lancet. (2000) 355:1291–2. doi: 10.1016/S0140-6736(00)02107-3, PMID: 10776738

[33] Conner M Kirk SFL Cade JE Barrett JH. Why do women use dietary supplements? The use of the theory of planned behaviour to explore beliefs about their use. Soc Sci Med. (2001) 52:621–33. doi: 10.1016/S0277-9536(00)00165-9, PMID: 11206658

[34] Ma L. Relationships, favors and administrative burdens--another discussion of public administration in daily life [OL]. J Pub Admin. (2024) 1:16. doi: 10.16149/j.cnki.23-1523.20241216.005

[35] Figueira HA Figueira OA Corradi-Perini C Martínez-Rodríguez A Figueira AA Da Silva CRL. A descriptive analytical study on physical activity and quality of life in sustainable ageing. Sustain For. (2021) 13:5968. doi: 10.3390/su13115968

[36] Kim S-Y. A study on the leisure sports participation behavior of the elderly through comparative analyses by age: focusing on leisure participation constraints and Price sensitivity. Behav Sci. (2024) 14:803. doi: 10.3390/bs14090803, PMID: 39336018 PMC11429335

[37] Zhao Y Hu Y Smith JP Strauss J Yang G. Cohort profile: the China health and retirement longitudinal study (CHARLS). Int J Epidemiol. (2014) 43:61–8. doi: 10.1093/ije/dys203, PMID: 23243115 PMC3937970

[38] Gong J Wang G Wang Y Chen X Chen Y Meng Q. Nowcasting and forecasting the care needs of the older population in China: analysis of data from the China health and retirement longitudinal study (CHARLS). Lancet Public Health. (2022) 7:e1005–13. doi: 10.1016/S2468-2667(22)00203-1, PMID: 36423656 PMC9741660

[39] Gao K Cao L-F Ma W-Z Gao Y-J Luo M-S Zhu J. Association between sarcopenia and cardiovascular disease among middle-aged and older adults: findings from the China health and retirement longitudinal study. eClinicalMedicine. (2022) 44:101264. doi: 10.1016/j.eclinm.2021.101264, PMID: 35059617 PMC8760427

[40] Wang Y Nie J Ferrari G Rey-Lopez JP Rezende LFM. Association of physical activity intensity with mortality: a national cohort study of 403 681 US adults. JAMA Intern Med. (2021) 181:203–11. doi: 10.1001/jamainternmed.2020.6331, PMID: 33226432 PMC7684516

[41] Li Y Cui M Pang Y Zhan B Li X Wang Q. Association of physical activity with socio-economic status and chronic disease in older adults in China: cross-sectional findings from the survey of CLASS 2020 after the outbreak of COVID-19. BMC Public Health. (2024) 24:37. doi: 10.1186/s12889-023-17492-9, PMID: 38166980 PMC10762973

[42] Bosdriesz JR Witvliet MI Visscher TL Kunst AE. The influence of the macro-environment on physical activity: a multilevel analysis of 38 countries worldwide. Int J Behav Nutr Phys Act. (2012) 9:110. doi: 10.1186/1479-5868-9-110, PMID: 22967164 PMC3490943

[43] Van Tuyckom C. Macro-environmental factors associated with leisure-time physical activity: a cross-national analysis of EU countries. Scand J Public Health. (2011) 39:419–26. doi: 10.1177/1403494810396553, PMID: 21273227

[44] Wang M Wen X Zhang Y Jiang C Wang F. Is economic environment associated with the physical activity levels and obesity in Chinese adults? A cross-sectional study of 30 regions in China. BMC Public Health. (2017) 17:701. doi: 10.1186/s12889-017-4699-4, PMID: 28899367 PMC5596845

